# Improving the Efficiency of Adventitious Shoot Induction and Somatic Embryogenesis via Modification of WUSCHEL and LEAFY COTYLEDON 1

**DOI:** 10.3390/plants9111434

**Published:** 2020-10-25

**Authors:** Miho Ikeda, Mikiya Takahashi, Sumire Fujiwara, Nobutaka Mitsuda, Masaru Ohme-Takagi

**Affiliations:** 1 Graduate School of Science and Engineering, Saitama University, Saitama 338-8570, Japan; retrospective.mirai@gmail.com (M.T.); mtakagi@mail.saitama-u.ac.jp (M.O.-T.); 2 Bioproduction Research Institute, National Institute of Advanced Industrial Science and Technology (AIST), Central 6, Tsukuba 305-8566, Japan; fujiwara-s@aist.go.jp (S.F.); nobutaka.mitsuda@aist.go.jp (N.M.)

**Keywords:** somatic embryogenesis, adventitious shoot formation, transcription factor, stem cell

## Abstract

The induction of adventitious organs, such as calli, shoots, and somatic embryos, in tissue culture is a useful technique for plant propagation and genetic modification. In recent years, several genes have been reported to be adventitious organ inducers and proposed to be useful for industrial applications. Even though the Arabidopsis (*Arabidopsis thaliana*) *WUSCHEL* (*WUS*) and *LEAFY COTYLEDON 1* (*LEC1*) genes can induce adventitious organ formation in Arabidopsis without phytohormone treatment, further improvement is desired. Here, we show that modifying the transcriptional repression/activation activities of WUS and LEC1 improves the efficiency of adventitious organ formation in Arabidopsis. Because WUS functions as a transcriptional repressor during the induction of adventitious organs, we fused it to an artificial strong repression domain, SUPERMAN REPRESSION DOMAIN X (SRDX). Conversely, we fused the strong transcriptional activation domain VP16 from herpes simplex virus to LEC1. Upon overexpression of the corresponding transgenes, we succeeded in improving the efficiency of adventitious organ induction. Our results show that the modification of transcriptional repression/activation activity offers an effective method to improve the efficiency of adventitious organ formation in plants.

## 1. Introduction

Induction of calli, adventitious shoots, and somatic embryos from somatic cells is important for plant propagation and transformation. Plant somatic cells tend to have a high potential for regeneration via dedifferentiation and formation of adventitious shoots and somatic embryos under suitable tissue culture conditions, which are generally determined by light quality, temperature, and phytohormone concentrations. However, in many plant species, it is challenging to find suitable tissue culture conditions for plant regeneration from somatic cells. Various genes have been reported to be involved in adventitious organ formation processes, such as somatic embryogenesis and shoot and callus formation. For instance, *WUSCHEL* (*WUS*) and *LEAFY COTYLEDON 1* (*LEC1*) are well documented for their role in somatic embryo induction [1,2].

WUS is a bifunctional homeodomain transcription factor that mainly acts as a repressor [3] and plays an important role in maintaining the pluripotent stem cell identity of the shoot and flower meristems [4,5]. Ectopic overexpression of Arabidopsis (*Arabidopsis thaliana*) *WUS* induces dedifferentiation of somatic cells, resulting in the formation of adventitious shoots and somatic embryos in Arabidopsis [1,6] as well as in tobacco (*Nicotiana tabacum*) [7], robusta coffee (*Coffea canephora*) [8], and cotton (*Gossypium hirsutum*) [9,10]. Furthermore, overexpression of maize (*Zea mays*) *WUS2* improves the transformation efficiency of monocots, such as rice (*Oryza sativa*), sorghum (*Sorghum bicolor*), and maize [11,12]. These reports indicate that the function of *WUS* orthologs is widely conserved across many plant species, including both dicotyledonous and monocotyledonous plants.

*LEC1* encodes a subunit of the NUCLEAR FACTOR-Y (NF-Y) transcriptional activator, which regulates gene expression during seed development [13,14]. *LEC1* and its orthologs are expressed during somatic embryogenesis in Arabidopsis, maize, carrot (*Daucus carota* subsp. *sativus*), European larch (*Larix decidua*), and cassava (*Manihot esculenta*), [15,16,17,18,19], indicating that *LEC1* also acts as a regulator of somatic embryogenesis in many plant species.

Given the abovementioned characteristics, *WUS* and *LEC1* would constitute useful genetic tools to improve plant propagation and transformation in various plant species. However, the native forms of Arabidopsis WUS and LEC1 are not sufficient to induce adventitious organ formation by themselves in several plant species. For example, robusta coffee requires phytohormone treatment in addition to the ectopic expression of *WUS* for somatic embryo formation [8]. Similarly, the ectopic expression of Arabidopsis *LEC1* alone does not induce somatic embryogenesis or shoots in transgenic tobacco plants [20].

We reasoned that a detailed analysis of Arabidopsis WUS and LEC1 molecular functions might yield some clues that would allow us to enhance their ability to induce adventitious organogenesis. We have previously discovered and reported that WUS has two functional domains: the WUS-box and the ethylene-responsive element binding factor-associated amphiphilic repression (EAR)-like motif [3]. Although both domains show strong repressive activity in transient expression assays, the biological functions of each domain are different [3]. We have shown that the WUS-box is a bifunctional domain, behaving as an activator domain for the induction of *AGAMOUS* (*AG*) expression and acting as a repressor domain for the suppression of the expression of type-A *ARABIDOPSIS RESPONSE REGULATOR* (*ARR*) genes [3]. The WUS-box might also be important for the ability of Arabidopsis WUS to induce adventitious organogenesis. The mutant WUS protein, WUSm1, which carries a mutated version of the WUS-box, appeared to have a reduced ability to induce adventitious organs [3]. The fusion of the artificial repression domain SUPERMAN REPRESSION DOMAIN X (SRDX) [21] to WUSm1, however, rescued the function of WUSm1 for the induction of adventitious organ formation, while fusion of the VP16 activation domain [22] to WUSm1 (WUSm1–VP16) failed to complement the same phenotypes [3]. These previous findings indicate that the WUS-box of ectopically expressed WUS acts as a repressor domain during the induction of adventitious organ formation [3].

In this report, we show that it is possible to enhance the capacity of Arabidopsis WUS and LEC1 to induce adventitious organ formation by fusing them to the SRDX repression domain and the VP16 activation domain, respectively. Our results indicate that modification of the transcriptional repression/activation activity of these transcription factors is an effective strategy to improve adventitious organ formation in plants.

## 2. Results

### 2.1. The WUS-Box Is the Main Functional Domain for Adventitious Organ Formation

**Figure 1:**
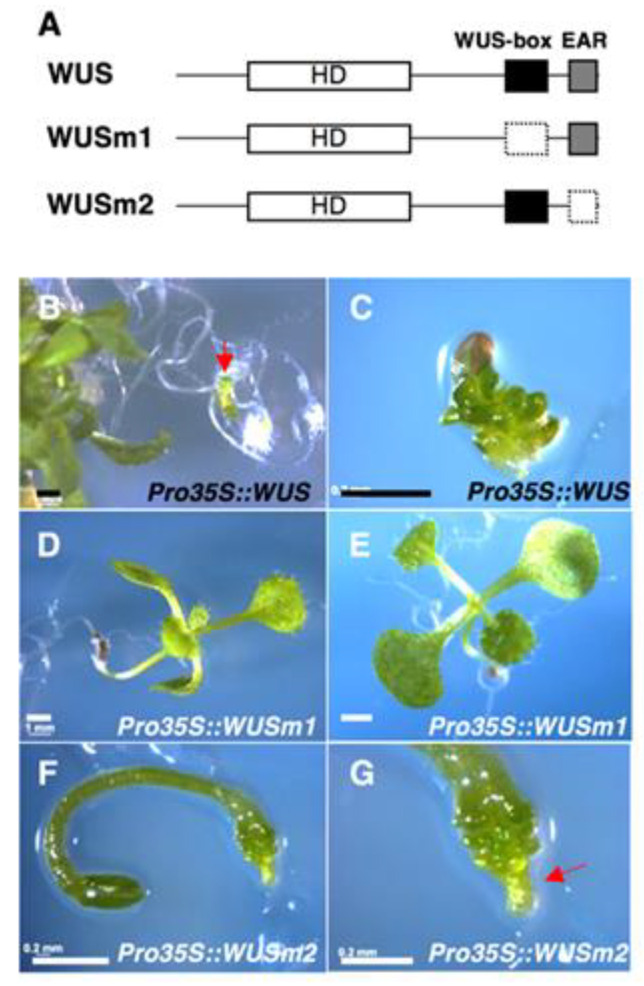
Phenotypes of transgenic seedlings ectopically accumulating modified WUSCHEL (WUS) proteins. (**A**) Schematic representation of the modified WUS proteins. The WUS-box (black box) or the ethylene-responsive element binding factor-associated amphiphilic repression (EAR) motif (gray box) of the WUS protein were mutated (indicated by a white box) in WUSm1 and WUSm2, respectively. (**B**,**C**) Phenotypes of *35Spro:WUS* primary transformants. One-month-old plant that formed somatic embryos (SE) on the roots (**B**) and a seven-day-old seedling with the green ball phenotype (**C**). (**D**,**E**) Twelve-day-old *35Spro:WUSm1* seedlings. Normal phenotype (**D**) and defective shoot apical meristem phenotype (**E**). (**F**,**G**) Seven-day-old *35Spro:WUSm2* seedling (**F**) and magnified image of the root region of the seedling (**G**). Bars = 1 mm. Red arrows indicate the somatic embryo that formed on the root.

To enhance the ability of WUS to induce adventitious organ formation, we first assessed the function of the two WUS domains, WUS-box and EAR motif, for the induction of adventitious organogenesis. We analyzed the detailed phenotypes of transgenic seedlings ectopically accumulating modified WUS proteins harboring mutations in either domain (Figure 1A, Figure S1) [3]. The ectopic expression of the native *WUS* gene driven by the 35S promoter (*35Spro:WUS*) induced various abnormalities. A fraction of young seedlings looked normal during the seedling stage but later developed roots that bent in an unnatural direction, turned green, and then formed somatic embryos and adventitious shoots, as reported previously (Figure 1B) [1,6]. Other seedlings showed an abnormal appearance like a green ball (Figure 1C), which lost the primary shoot, roots, and hypocotyl, and formed numerous shoot meristems and/or somatic embryos. These observations indicate that the ectopic expression of *WUS* has two effects: one disturbs normal zygotic embryogenesis and the other confers stem cell identity to somatic cells in seedlings.

Seedlings expressing *35Spro:WUSm1*, which contains a mutation in the sequence encoding the WUS-box, showed a normal appearance (Figure 1D). This observation demonstrated that the WUSm1 variant had lost its function. Notably, some *35Spro:WUSm1* seedlings lost the shoot meristem just after the formation of several leaves (Figure 1E), a phenotype that resembles a WUS loss-of-function mutant (such as *wus-1*), which cannot maintain the stem cell population at the shoot apical meristem. This finding suggests that WUSm1 may function as a dominant negative molecule, disrupting the function of the endogenous wild-type protein (Figure 1E). These data show that the WUS-box from ectopically expressed WUS is necessary for the induction of adventitious organs and disturbing normal embryonic development.

The expression of *35Spro:WUSm2*, which encodes a WUS variant with a mutated EAR motif, induced an abnormal seedling phenotype as well. Most seedlings had cotyledons, a long hypocotyl, and a root meristem (Figure 1F), but the primary root meristem frequently formed additional shoots or somatic embryos (Figure 1G). This observation suggests that the root meristem is converted to a shoot or embryonic meristem in these transgenic lines. In addition, leaf formation from the primary shoot meristem was delayed in *35Spro:WUSm2* seedlings, suggesting that the shoot meristem is also defective. These morphological features indicate that WUSm2 still retains the ability normally seen with wild-type WUS to confer stem cell identity to somatic cells and interfere with normal embryonic development, albeit with a weaker activity. Thus, the EAR-like motif plays a role in WUS function, but its contribution appears to be minimal. From these data, we conclude that the WUS-box acts as the main functional domain of the WUS protein in terms of induction of adventitious organ formation and disturbance of normal embryonic development when ectopically expressed.

### 2.2. Fusion with an Artificial Repression Domain Enhances the Ability of WUS to Induce Adventitious Organ Formation

**Figure 2:**
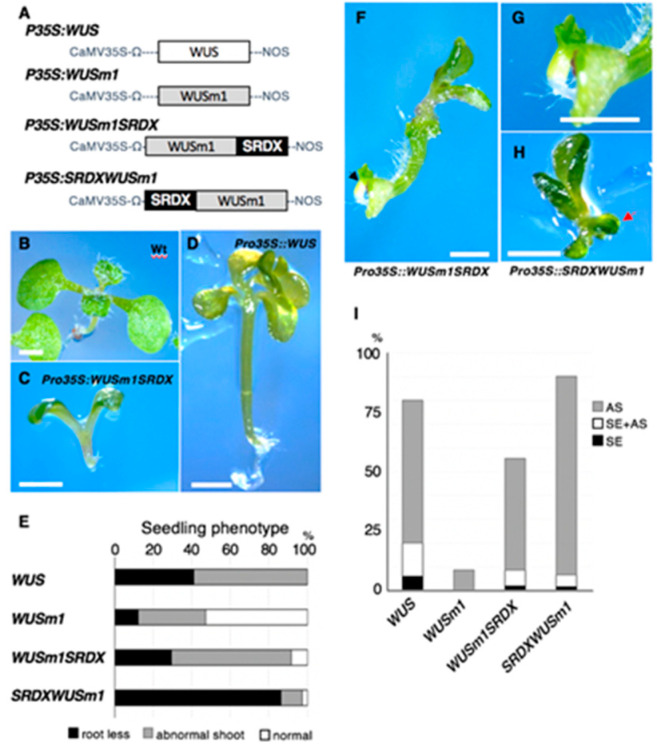
Domain complementation analysis by fusing the SUPERMAN REPRESSION DOMAIN X (SRDX) repressor domain to the WUSm1 mutant protein. (**A**) Schematic representation of the constructs used for morphological analysis. White, gray, and black boxes indicate the native *WUS* gene, *WUSm1*, and the fragment encoding the SRDX repressor domain, respectively. (**B**–**D**) Seven-day-old seedlings showing the normal phenotype ((**B**): wild-type seedling), root-less phenotype ((**C**): *35Spro:WUSm1–SRDX*), and abnormal cotyledon phenotype ((**D**): *35Spro:WUS*). Bar = 1 mm. (**E**) Frequency of each phenotype in 14-day-old primary transformants: normal seedling (white column), seedling with abnormal shoot (gray column), and root-less phenotype (black column). *n* > 34. (**F**–**H**) Seedlings showing somatic embryogenesis ((**F**): seven-day-old *35Spro:WUSm1–SRDX* seedling) and adventitious shoot formation ((**H**): six-day-old *35Spro:SRDX–WUSm1* seedling). A magnified image of a somatic embryo that formed on the root in (**F**) is shown in (**G**). Red and black arrows indicate the adventitious shoot and somatic embryo, respectively. Bar = 1 mm. (**I**) The rate of adventitious shoot (AS) and somatic embryo (SE) formation in 29-day-old primary transformants. *n* > 35.

In our previous report, we showed that the WUS-box from ectopically expressed WUS acted as a repression domain in the induction of adventitious organ formation and disturbance of normal embryonic development [3]. The fusion of the artificial repression domain SRDX [21] to the C- or N-terminus of WUSm1 (WUSm1–SRDX or SRDX–WUSm1) (Figure 2A) successfully rescued the function of WUSm1 protein in terms of both the induction of adventitious organ formation and disturbance of normal embryonic development. Although both *35Spro:WUSm1–SRDX* and *35Spro:SRDX–WUSm1* plants showed abnormal embryogenic development and adventitious organ formation, the frequency at which they did so was different between these two WUSm1 fusion constructs.

In this study, we aimed to clarify the frequency of adventitious organ formation in each type of transgenic plants. We roughly classified the phenotypes of 14-day-old seedlings from primary transformants expressing either *35Spro:WUSm1–SRDX*, *35Spro:SRDX–WUSm1*, *35Spro:WUS* (positive control), or *35Spro:WUSm1* (negative control) into three types: normal seedling, seedling with abnormal shoot, and seedling without a root (Figure 2B–D). Abnormal shoot phenotypes included an abnormal number of cotyledons, cotyledons with a rough surface, and delayed leaf development. The phenotypes of seedlings without root included seedlings with no root meristem and seedlings with delayed rooting. Seedlings exhibiting both abnormal shoot and abnormal root phenotypes were classified as seedlings without a root because the abnormal root phenotype was more conspicuous than the abnormal shoot phenotype. When we scored 14-day-old seedlings expressing the positive control *35Spro:WUS*, we observed a root-less and abnormal shoot phenotype in 41% and 59% of the seedlings, respectively (Figure 2E). By contrast, 53% of the 14-day-old seedlings expressing the negative control *35Spro:WUSm1* were normal, while 12% and 35% showed no root and abnormal shoot phenotypes, respectively (Figure 2E). In *35Spro:WUSm1–SRDX* seedlings, 29% and 62% showed no root and abnormal shoot phenotypes, respectively, while the remaining 8% looked normal. These data showed that the effect of the *35Spro:WUSm1–SRDX* construct was weaker than that of the *35Spro:WUS* construct but stronger than that of the *35Spro:WUSm1* construct. By contrast, more than 86% of the 14-day-old seedlings expressing the *35Spro:SRDX–WUSm1* construct did not develop roots (Figure 2E), indicating that the effect of the *35Spro:SRDX–WUSm1* construct on embryonic development was even stronger than that of the *35Spro:WUS* construct.

Next, we analyzed the effect of each modified WUS protein on the induction of adventitious shoot formation and somatic embryogenesis. In some cases, somatic embryos and adventitious shoots formed on the roots of the transgenic seedlings immediately after germination (six days after sowing) (Figure 2F,G). Further observations demonstrated that roughly 80%, 8%, 55%, and 90% of the primary transformants expressing *35Spro:WUS* (positive control), *35Spro:WUSm1* (negative control), *35Spro:WUSm1–SRDX*, and *35Spro:SRDX–WUSm1* formed adventitious shoots and/or somatic embryos, respectively, after 28 days of growth since sowing (Figure 2H). These data indicate the possible enhancement of the adventitious organ formation ability of WUSm1 by the addition of the SRDX repression domain to its N-terminus. It should be noted that the *35Spro:SRDX–WUSm1* transgenic T_1_ plants had a higher rate of adventitious shoot formation and a slightly lower rate of somatic embryo formation compared to *35Spro:WUS* transgenic T_1_ plants (Figure 2H). These data indicate that domain modification may also affect the type of adventitious organ induced by each transgene.

### 2.3. Fusion of an Artificial Activation Domain Enhances the Ability of LEC1 to Induce Callus Formation

**Figure 3:**
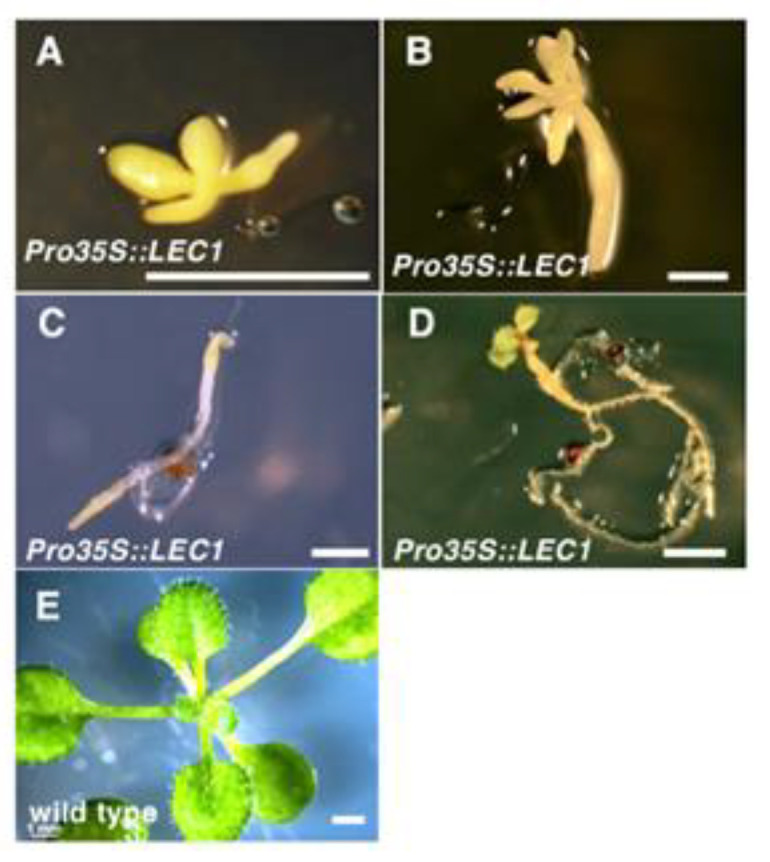
Phenotypes of transgenic seedlings ectopically accumulating modified LEAFY COTYLEDON 1 (LEC) proteins. (**A**–**E**) Fourteen-day-old *35Spro:LEC1* seedlings (**A**–**D**) and wild-type seedlings (**E**) grown under normal light. Bars = 1 mm.

LEC1 is also an inducer of adventitious organ formation as ectopic expression of *LEC1* induces somatic embryogenesis [2]. However, the phenotype of *35Spro:LEC1* transgenic seedlings is different from that of *35Spro:WUS* transgenic plants. *35Spro:LEC1* seedlings developed two small cotyledons but normal-sized hypocotyls and root tips, although they showed delayed growth such as delayed cotyledon expansion, root elongation, and leaf formation (Figure 3A–E). Notably, *35Spro:LEC1* seedlings retained an opaque pale-yellow color similar to that of a mature embryo under normal light and did not turn green for more than two weeks (Figure 3A–D). Wild-type seedlings also showed the opaque pale-yellow phenotype immediately after germination, but soon after the cotyledons turned green, and the opaque pale-yellow color was lost even in the hypocotyl and root tip. Some *35Spro:LEC1* transgenic seedlings with a milder phenotype showed slight root elongation and/or the formation of small, yellow, trichome-less leaves (Figure 3B,C), suggesting that shoot and root apical meristems were present. However, the root tip of *35Spro:LEC1* seedlings sometimes retained the embryonic pale-yellow color even after elongation (Figure 3C). These observations suggest that *35Spro:LEC1* transgenic seedlings are not impaired in embryonic development, but they may have delayed/altered germination and may maintain embryonic identity for several weeks.

**Figure 4:**
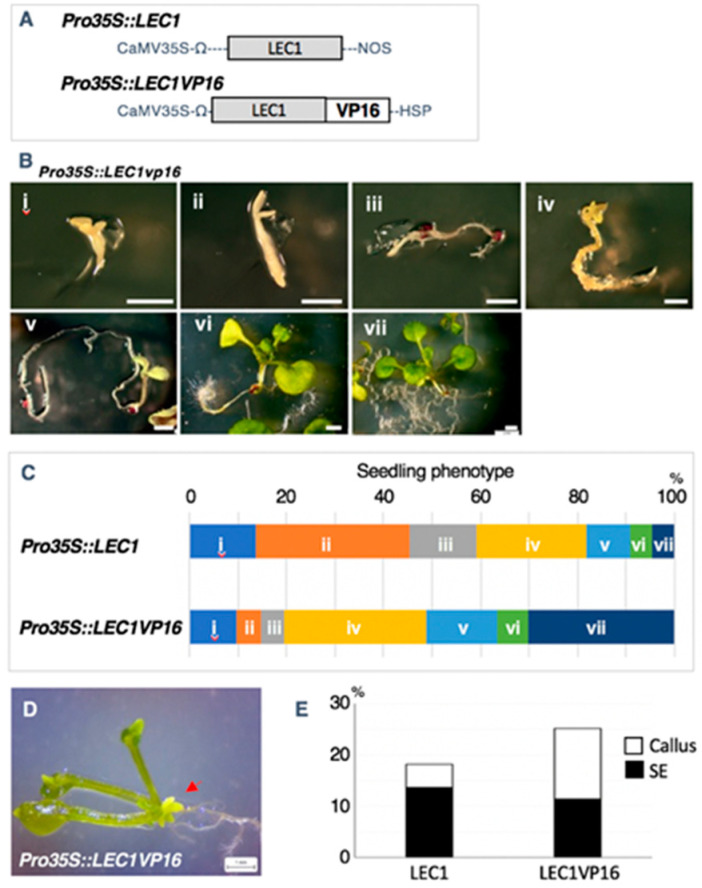
Analysis of domain fusion to LEC1. (**A**) Schematic representation of the constructs used for morphological analysis. Gray and white boxes indicate the native *LEC1* gene and the *VP16* activation domain, respectively. CaMV35S, Ω, NOS, and *HSP* indicate the Cauliflower mosaic virus 35S promoter, omega enhancer sequence, *NOS* terminator sequence, and heat shock protein terminator sequence, respectively. (**B**) Seven phenotypes of 14-day-old *35Spro:LEC1–VP16* seedlings. Opaque pale-yellow seedling without shoot and root development (i), seedling with hypocotyl elongation (ii), seedling with root elongation (iii), small trichome-less leaves (iv), green seedling with growth delay (v), green seedling with abnormal thick root (vi), and normal seedling (vii). (**C**): The ratio of each phenotype in 18-day-old primary transformants. *n* > 22. (**D**) Three-week-old *35Spro:LEC1–VP16* transgenic plants that formed a somatic embryo. Red arrow indicates the somatic embryo. (**E**) Percentage of calli and SE formation in 25-day-old primary transformants. *n* > 22. Bars = 1 mm.

LEC1 has no known repression motif and is therefore likely to be an activator. Accordingly, we chose to add the strong VP16 activation domain [22] to the C-terminus of LEC1 in the hope of enhancing the ability of LEC1 to induce adventitious organ formation (Figure 4A). We then ectopically expressed it under the control of the cauliflower mosaic virus (CaMV) 35S promoter and the *Heat Shock Protein* (*HSP*) terminator (*35Spro:LEC1–VP16*), which is reported to be an effective terminator supporting increased levels of expression [23]. We classified the phenotypes of primary transformants for *35Spro:LEC1–VP16* and *35Spro:LEC1* (control) into seven types, from severe to mild: opaque pale-yellow seedling without shoot or root development (Figure 4B, type i); yellow seedling with hypocotyl elongation (Figure 4B, type ii); yellow seedling with root elongation (Figure 4B, type iii); yellow seedling with small, trichome-less leaves (Figure 4B, type iv); green seedling with growth delay (Figure 4B, type v); green seedling with abnormal thick root (Figure 4B, type vi); and normal seedling (Figure 4B, type vii). More than 80% of seedlings from 18-day-old control plants expressing *35Spro:LEC1* exhibited an opaque yellow color, of which 13.6% failed to grow shoots or roots (Figure 4C). By contrast, about 50% of 18-day-old plants expressing *35Spro:LEC1–VP16* showed yellow color, of which 9.8% failed to grow shoots or roots (Figure 4B). The percentage of seedlings with a normal phenotype among the *35Spro:LEC1* and *35Spro:LEC1–VP16* primary transformants was 4.5% and 30.0%, respectively (Figure 4B). These data showed that the phenotype of *35Spro:LEC1–VP16* transgenic seedlings was unexpectedly weaker than that of *35Spro:LEC1* seedlings in terms of maintaining embryonic identify after germination.

Next, we analyzed the effect of a modified *LEC1* construct on the induction of adventitious organ formation. Twenty-five days after sowing, around 10% of primary transformants expressing either *35Spro:LEC1* or *35Spro:LEC1–VP16* formed somatic embryos; however, a higher proportion of *35Spro:LEC1–VP16* transgenic T_1_ plants displayed callus formation relative to *35Spro:LEC1* plants (Figure 4D,E). These data suggest that the addition of the VP16 activation domain or the use of the *HSP* terminator may have enhanced the adventitious organ formation ability of LEC1 but with a lower ability to maintain embryonic identity after germination.

## 3. Discussion

To promote the activity of WUS and LEC1 for the induction of adventitious organ formation, such as somatic embryos, adventitious shoots, and calli, from somatic cells, it is essential to understand their underlying functions. WUS induces ectopic stem cell identity [1,24], and somatic embryogenesis and shoot formation occur from roots of transgenic plants ectopically expressing *WUS* [1]. The *35Spro:WUS* seedlings sometimes lacked various organs, such as roots, cotyledons, and hypocotyls (Figure 1), suggesting that organ formation during the zygotic embryogenesis of *35Spro:WUS* transgenic plants had been disturbed. We observed superfluous cell division and adventitious organ formation in the roots of *35Spro:WUS* seedlings immediately after germination (Figure 1). The ectopically expressed *WUS* might induce the formation of ectopic pluripotent stem cells in root cells of *35Spro:WUS* zygotic embryos during embryogenesis, from which adventitious organs will form after germination.

Because the seedlings of *35Spro:WUS* formed both adventitious shoots and somatic embryos (Figure 2), we hypothesize that ectopically expressed *WUS* can induce stem cell identity but cannot control whether the cell differentiates into a shoot or an embryo. Lenhard et al. reported that the ectopic expression of *WUS* induces the expression of the stem cell marker gene *CLAVATA3* (*CLV3*), but does not induce the shoot meristem marker genes *KNOTTED-LIKE* (*KNAT*)*1* and *KNAT2* [24], which supports our assumption. According to this hypothesis, two steps are needed for the induction of somatic embryos or adventitious shoots: (1) the acquisition of ectopic stem cell identity and (2) the regulation of cell differentiation of the induced ectopic stem cells. WUS appears to regulate the first step. The accumulation of a modified WUS with an artificial SRDX transcriptional repressor domain at its N-terminus (SRDX–WUSm1) increased the rate of adventitious organ formation, including adventitious shoots and somatic embryos (Figure 2) [3]. This finding indicates that the SRDX domain enhances the ability of WUS to induce ectopic stem cell identity. However, the position of the SRDX repressor domain is important because its addition to the C-terminus was not effective (Figure 2E).

The ectopic expression of *SRDX–WUSm1* was effective in inducing adventitious shoot formation but not somatic embryogenesis (Figure 2E). We have previously demonstrated that the repression activity of WUS is important to repress the expression of type-A *ARRs*, which suppresses cytokinin signaling [3]. The SRDX strong artificial repressor domain fused to the N terminus of WUSm1 might confer additional strong repression activity to the fusion protein and increase indeterminate shoot formation via the promotion of cytokinin signaling. To navigate the differentiation fate of ectopic stem cells induced by WUS, coexpression of additional factors that control cell differentiation may be effective. *SHOOT MERISTEMLESS* (*STM*) and *ENHANCER OF SHOOT REGENERATION* (*ESR*) can be considered as candidates to enhance adventitious shoot formation. Ectopically expressed *STM* was found to induce the expression of the shoot meristem marker genes *KNAT1* and *KNAT2* [24,25], and the ectopic expression of *ESR1* and *ESR2* induced shoot regeneration from various explants [26,27].

*LEC1*, *LEC2*, and *BABY BOOM* (*BBM*), which induce somatic embryogenesis when ectopically expressed in plants [2,19,28,29,30,31,32], may be considered as candidates to enhance somatic embryo induction. Our detailed observations revealed that the seedling phenotypes of *35Spro:LEC1* primary transformants were completely different from that of *35Spro:WUS* seedlings. The seedlings expressing *35Spro:LEC1* had all the organs that constitute a normal seedling (cotyledons, hypocotyl, and root); however, the seedlings retained their embryonic opaque yellow color for several weeks under normal light (Figure 3). Tobacco seedlings ectopically expressing Arabidopsis *LEC1* exhibited embryonic characteristics for 20 days after germination [20]. These findings suggest that *35Spro:LEC1* seedlings maintain the embryonic identity after germination and fail to switch to postgermination growth. Although it is difficult to clarify whether ectopic expression of *LEC1* induces de novo embryonic identity in seedlings postgermination or simply maintains the original embryonic identity, LEC1 may be a candidate for enhancing somatic embryogenesis induction. We also attempted to enhance the activity of LEC1; however, we failed to increase the frequency of somatic embryogenesis induction by modifying LEC1 (Figure 4). Instead of LEC1, BBM may be a better candidate because coexpression of maize *BBM* and *WUS2* was found to promote somatic embryogenesis in maize and sorghum [33].

In this study, we attempted to enhance the ability of *WUS* and *LEC1* to induce the formation of adventitious organs by modifying the transcriptional repression or activation domains of their corresponding proteins. The total rate of adventitious organ formation, including somatic embryos, adventitious shoots, and calli, increased in the transgenic plants ectopically expressing modified *WUSm1* or *LEC1–VP16*. These transgenes may now be utilized for the induction of adventitious organ formation in other plant species, including dicotyledonous and monocotyledonous plants. As a next step, it will be important to establish a method to control the redifferentiation fate of ectopically induced stem cells.

## 4. Materials and Methods

### 4.1. Growth and Transformation of Plants

Arabidopsis (*Arabidopsis thaliana*) accession Columbia-0 (Col-0) was used in all experiments. Plants were grown on soil at 22 °C under a 16 h light photoperiod. Transformation of Arabidopsis was performed by the floral-dip method [34]. For observation of the phenotype of each transgenic seedling, the seeds that surface-sterilized with 1% NaClO solution were sown on solid half-strength Murashige and Skoog (MS) selection medium containing 30 μg/mL hygromycin and incubated at 21 °C under continuous light.

### 4.2. Construction of Plasmids

The coding regions of *WUS* and *LEC1* and mutated *WUS* (*WUSm2*) were PCR-amplified from an Arabidopsis cDNA library with the appropriate primers (Table S1). To construct the *WUSm1* constructs, mutated *WUSm1* was separately amplified with two pairs of forward and reverse primers (At2g17950N and WUSm2R, WUSm2F, and 2g19750ox) (Table S1). The constructs for overexpression and SRDX fusion to the C-terminal and N-terminal regions for *WUSm1*, wild-type *WUS*, and *LEC1* were based on the vectors named as pro35SG [35], pro35SSRDXG [36], and pro35S_M_SRDXG [3], respectively. After confirmation of the insert sequence, each transgene cassette was transferred into the binary destination vector pBCKH [36], which contained a hygromycin resistance gene, by Gateway LR cloning (Thermo Fisher Scientific, Waltham, MA, USA). For *35Spro:LEC1–VP16* construction, the *LEC1* fragment flanked by Gateway attB1 and attB2 sequences (Thermo Fisher Scientific) at the 5′ and 3′ ends, respectively, was cloned into pDONR207 (Thermo Fisher Scientific) and introduced into pDEST_35S_VP16_HSP_GWB5 [37] (Fujiwara et al. 2014) by Gateway LR clonase II cloning (Thermo Fisher Scientific).

### 4.3. Microscopy Observations

For the observation of each transgenic seedling, ZEISS Stemi 305 (Carl Zeiss) and SZ2 (Olympus) microscopes were employed.

## Supplementary Materials

The following are available online at https://www.mdpi.com/2223-7747/9/11/1434/s1, Figure S1: Amino acid sequence of the WUS-box and the carboxy-terminal EAR-like motif. Mutations introduced into the WUS-box (m1) and EAR-like motif (m2) are indicated by arrowheads. Table S1: Oligonucleotides used in this study.
