# Supplementary material for: Improving the Efficiency of Adventitious Shoot Induction and Somatic Embryogenesis via Modification of WUSCHEL and LEAFY COTYLEDON 1
Source: Plants (Basel). 2020 Oct 25;9(11):1434. doi: 10.3390/plants9111434 (PMC7692810; doi:10.3390/plants9111434)
Supplement: Supplementary file 1 [file plants-09-01434-s001.zip › plants-953969-supplementary/plants-953969-supplementary.docx]

Supplementary Materials:

| 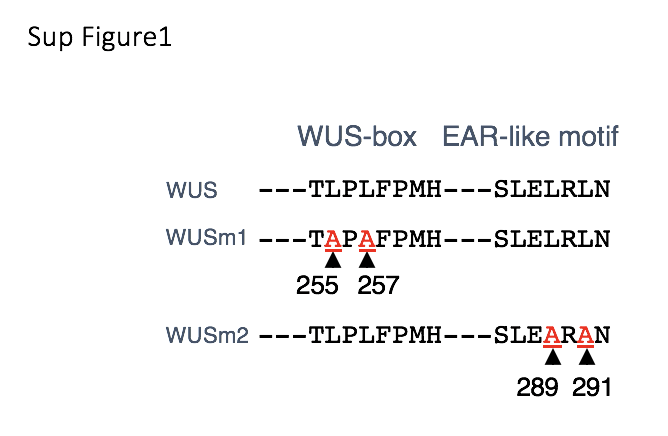 |  |
| --- | --- |

**Sup Figure 1.** Amino acid sequence of the WUS-box and the carboxy-terminal EAR-like motif.Mutations introduced into the WUS-box (m1) and EAR-like motif (m2) are indicated by arrowheads.
